# A Novel Trademark Image Retrieval System Based on Multi-Feature Extraction and Deep Networks

**DOI:** 10.3390/jimaging8090238

**Published:** 2022-09-02

**Authors:** Sandra Jardim, João António, Carlos Mora, Artur Almeida

**Affiliations:** 1 Smart Cities Research Center, Polytechnic Institute of Tomar, 2300-313 Tomar, Portugal; 2 Techframe-Information Systems, SA, 2785-338 São Domingos de Rana, Portugal

**Keywords:** image retrieval, content-based image retrieval (CBIR), deep convolutional neural networks (DCNN), trademark, combined multiple features

## Abstract

Graphical Search Engines are conceptually used in many development areas surrounding information retrieval systems that aim to provide a visual representation of results, typically associated with retrieving images relevant to one or more input images. Since the 1990s, efforts have been made to improve the result quality, be it through improved processing speeds or more efficient graphical processing techniques that generate accurate representations of images for comparison. While many systems achieve timely results by combining high-level features, they still struggle when dealing with large datasets and abstract images. Image datasets regarding industrial property are an example of an hurdle for typical image retrieval systems where the dimensions and characteristics of images make adequate comparison a difficult task. In this paper, we introduce an image retrieval system based on a multi-phase implementation of different deep learning and image processing techniques, designed to deliver highly accurate results regardless of dataset complexity and size. The proposed approach uses image signatures to provide a near exact representation of an image, with abstraction levels that allow the comparison with other signatures as a means to achieve a fully capable image comparison process. To overcome performance disadvantages related to multiple image searches due to the high complexity of image signatures, the proposed system incorporates a parallel processing block responsible for dealing with multi-image search scenarios. The system achieves the image retrieval through the use of a new similarity compound formula that accounts for all components of an image signature. The results shows that the developed approach performs image retrieval with high accuracy, showing that combining multiple image assets allows for more accurate comparisons across a broad spectrum of image typologies. The use of deep convolutional networks for feature extraction as a means of semantically describing more commonly encountered objects allows for the system to perform research with a degree of abstraction.

## 1. Introduction

The accelerated global technological advance that we are currently witnessing is reflected in an increasing digitization of all types of information, from simple textual information to more complex information, such as images or videos. At the same time, storage systems are increasingly used, resulting in extensive databases both for general use and for specific use in certain professional areas. If, on the one hand, the amount of information available today is much greater and easy to access, on the other hand, with the increase in the size of databases, it becomes a challenge to exactly identify the information sought. In this context, the accuracy and effectiveness of search engines play a key role, especially when applied in professional areas, such as medical diagnostics or industrial property.

Image retrieval, a research area of Information Retrieval [1] of great scientific interest since the 1970s, is a widely studied image matching problem where, for a given query image, similar images are retrieved from a database [2,3]. Conventional approaches to image research systems are based on textual annotations that keep crucial content about the image and that are used in carrying out the research. However, these annotations are often lost as a result of image compression or human error. Additionally, due to the wide variety of image annotation standards in different professional areas, the definition of a common ontology is not always consensual. Earlier studies include manual annotation of images using keywords and searching by text [4]. Content Based Image Retrieval (CBIR) [5] was proposed in the 1990s to overcome the difficulties of text-based image retrieval using manual annotation of images based on subjective human perception and the time and labor requirements of annotation [6]. CBIR refers to the fully automatic process of obtaining images that are relevant to a query image from a large collection based on their visual content [7,8,9].

Given the feature representations of the images to be searched and the query image, the output of the CBIR procedure includes a search in the feature space in order to retrieve a ranked set of images in terms of similarity (e.g., cosine similarity) to the query image representation.

A key issue associated with CBIR is to extract meaningful information from raw data in order to eliminate the so-called semantic gap [3,10] that leads to irrelevant image retrieval. The semantic gap refers to the difference between the low-level representations of images and their higher level concepts (perception) [10]. Over the past three decades, this gap has been the focus of numerous studies [11].

In recent years, in the areas of application of CBIR systems, the use of large databases has become feasible with the increase in the capacity of processors and the decrease in the price of memories, resulting in an increasingly pressing need for effective image recovery systems. Industrial property [6,12,13,14,15], medical imaging [16,17,18,19,20,21], criminal investigation [22,23,24] and online image search engines [25] are some examples of the areas in which CBIR techniques are used.

The application of CBIR systems to the area of Industrial Property is particularly challenging since it is intended to model the human perception of abstract images and to identify those that have a similar perception. The main characteristic of images that determines human perception is shape [26]. For instance, for a search in a logo database for an image that contains a circle in a triangle, a logo that contains those elements deformed, but in the same layout, is considered more similar than a logo that contains exactly same elements, but in different positions [27]. Thus, a system of automatic recovery of images of this context in a database, in addition to assessing their overall form, must consider possible variations of the elements, such as rotation, scaling, inversion, partial occlusion and noise.

Despite the remarkable progress on theory, technology and application in the past decade associated with CBIR systems, several problems related to the effectiveness and reliability of this type of systems are still to be solved [28,29,30,31,32]. This fuels the interest of researchers, being the most research concentrated on the emergence of big data and on the use of deep learning techniques to improve accuracy, often at the expense of increasing running time [33]. There is still a clear gap between the proposed solutions and the real needs of the market, particularly in the area of Industrial Property [27]. The limitations of these solutions are mainly related to three criteria that a trademark image recovery system must fulfill [27].

- The system must consider all possible interpretations of a trademark image. It is consensual in the literature that the process of human perception of an abstract image is complex. However, most of the automation solutions of these systems are based on low-level characteristics, such as shape, color or texture [6,14]. Several approaches have been proposed for the interpretation of the images, from their analysis as a whole to the analysis by segments, from the analysis of regions to the analysis by edges. However, with the exception of some projects, such as the System for Trademark Archival and Registration (STAR) [34], the solutions do not contemplate the multiple possibilities of interpreting an image.
- The system must be able to search large databases in a timely manner. Most of the solutions proposed so far have been studied with databases with just over 1000 images, and research efficiency has not been considered a development priority. However, the analysis of several low-level characteristics (color, shape and texture) and the respective variations (rotation, scaling, inversion or noise) require high computation times, which prove to be an impediment in the analysis of large databases. As such, there is an urgent need to improve the efficiency of these solutions to enable their application in a real context [30]. This efficiency improvement, absolutely necessary in view of the high number of records with which the system must work, requires the application of parallel processing techniques, segmenting the search into several sub-searches (for example, the division into 45 sub-searches, in accordance to the Nice Classification method), which must then be integrated and processed together after each sub-search is completed.
- Images very similar to the searched image cannot be omitted (zero tolerance). This is a fundamental requirement for trademark image recovery systems. Thus, for the application of these solutions in a real context, the systems must be thoroughly tested to ensure compliance with this criterion. It should be noted that these tests are essential, not only to ensure “zero tolerance”, but also to ensure that the amount of false positives is reduced enough to allow further analysis by humans, thus enabling the application of the respective systems in a real context of use.

This paper describes a novel image retrieval system designed to be used in the Industrial Property area, capable of providing highly accurate results regardless of the complexity and size of the dataset. The proposed method is based on a multi-phase implementation of different deep learning and image processing techniques whose functionality can vary depending on the system input, which can be one of three possible inputs. The proposed approach uses images’ signatures to provide a near exact representation of an image, with abstraction levels that allow the comparison with other signatures as a means to achieve a fully capable image comparison process. The system is designed to improve algorithm robustness in cases where the overall image disposition may not be similar, but there may be an individual component on the image which automatically flags it as plagiarism. The proposed system offers the ability to extensively filter search parameters, allowing the user to fine-tune the depth of search to perform. To overcome performance disadvantages related to multiple image searches due to the high complexity of image signatures, the proposed system incorporates a parallel processing block responsible for dealing with multi-image search scenarios. The system achieves image retrieval throughout a new similarity compound formula that accounts for all components of an image signature. From the results, it is possible to conclude that the developed approach performs image retrieval with high accuracy, showing that combining multiple image assets allows for more accurate comparisons across a broad spectrum of image typologies. Furthermore, the results show the benefit of using deep convolutional networks for feature extraction as a means of semantically describing more commonly encountered objects, allowing for the system to perform research with a degree of abstraction.

### Document Organization

This document is organized by sections as described. Section 2 describes related work and different state-of-the-art approaches used for content-based image retrieval systems. Section 3 describes the proposed framework and related details of the proposed system. In Section 4, results obtained by using the developed image retrieval system in a realistic use-case scenario are presented and discussed. This section also presents the performance of the proposed image retrieval system, as well as its comparison with other state-of-the-art proposals. Section 5 provides a brief concluding remark with conclusions and proposed future work.

## 2. Related Work

For the last 30 years, content-based image retrieval (CBIR) has been an active research area and a possible solution for implementing similar image retrieval systems from a large repository.

### 2.1. Conventional Feature Based Methods

The key step of a CBIR system is the representation of the image from which the critical features are extracted and transformed into a vector of fixed size, called the feature vector. These features are heuristically designed and can be categorized into global and local features. While earlier works focus on global features that describe the image content such as color, texture and shape [35,36,37,38,39,40], several works have been proposed on the direction of finding semantically richer image representations. Among the most effective are those that use the Fisher Vector descriptors [41], the Vector of Locally Aggregated Descriptors (VLAD) [42,43] or combine bag-of-words models [44] with local descriptors such as Scale-Invariant Feature Transform (SIFT) [45]. However, most of the CBIR systems are still based on the image’s low level features which are designed by assigning fixed or equal weights to all features [31,46,47]. Using mixed feature methodologies can potentially provide more successful results than a particular feature methodology [28].

Several techniques have been presented to build descriptors, such as the Scale Invariant Feature Transform (SIFT) [45], Histogram of Oriented Gradients (HOG) [48,49], Speeded-Up Robust Features (SURF) [50,51,52], Binary Robust Invariant Scalable Keypoints (BRISK) [53,54] and Maximally Stable Extremal Regions (MSER) [55], which have been applied for robust image searches by content. Several combinations and applications of these techniques have been presented in the literature. However, a totally effective solution is not yet known, since the techniques for building descriptors are not robust for all possible transformations [56]. Within these techniques, SIFT and SURF stand out. The first is more robust for rotations and scale changes and is also able to capture local margins of objects and shapes based on the distribution of intensity gradients [57]. This technique is particularly effective on images with simple backgrounds, representing them without noise. In images with complex backgrounds and with noise, the effectiveness of this technique decreases significantly [58]. The second technique, SURF, in contrast, has shown good results in representation and images with changes in lighting [59], being the respective most distinctive descriptor [60]. A study published in 2016 addressed the integration of these two techniques, having demonstrated the potential of their combination to increase the accuracy, efficiency and reliability of CBIR. The analysis and comparison of the results obtained were performed in Corel-1.5k, Corel-2k and Corel-1k [56].

Of the several techniques studied to support and complement the proposed solutions, it is important to highlight the Relevance Feedback (RF) interactive process [61]. This process has as its main purpose to approximate the two levels of characteristics used in the various approaches to assess the similarity of images, namely low-level characteristics and content characteristics. It is intended to provide the system with learning capacity based on user feedback. More specifically, this solution allows the user to classify and quantify the relevance of the results, serving this feedback to improve the research through machine learning techniques, such as the Naive Bayes method.

Despite the advances in deep learning methodologies and the trend of their application in the construction of CBIR systems, systems based on traditional approaches continue to be proposed.

Singh and Batra proposed a bi-layer CBIR system where the primitive image features of color, texture and shape are used [62]. The similarity between query image and dataset images is computed in two layers, the system being divided into two modules. In the first module, image features of the dataset images are extracted in the form of color, texture and shape, using a histogram on the HSV color space, a Gabor filter with five scales and six orientations, and the zernike moments, respectively. The second module performs the retrieval task which is further divided into two layers. In the first layer, the features shape and texture are used to compute the similarity of the query image to the images in the dataset. The indexes of most similar images, produced by pruning the non-relevant images based on similarity computed from the first layer, are the input to the second layer, where shape and color features of the query image are matched, and the most similar images are retrieved as output of the system. The authors evaluated the system on the image datasets COREL and GHIM-1k, achieving precisions ranging from 75% to 100% for different classes of the COREL dataset.

Another example of using low-level image features is proposed by Ashraf et al. [63]. As in [62], the authors proposed a technique of feature combination, using the color and texture features, which are extracted using Color Moments and Gabor Wavelet and Discrete Wavelet transform, respectively. The proposed system is divided into three phases that includes feature extraction, similarities match and performance evaluation. The authors evaluated the system on several image datasets, reporting average precisions of 87.5%, for COREL-1k; 86.33%, for COREL-1.5k; 79.83%, for GHIM-10k and 76.83%, for GHM-20k.

Recently, Fadaei proposed a Dominant Color Descriptor method, aiming to improve the CBIR systems accuracy [64]. The proposed method uses the Canny algorithm to extract the edges of the images, which are subsequently enlarged, using morphological operations, with higher weights being assigned to the pixels belonging to the edges, while pixels not belonging to the edges are assigned smaller weights. In this way, pixels in regions with low color variations are less weighted, and more informative pixels are more weighted in providing Dominant Color Descriptor features. The author shows the effectiveness of the method he proposes through the results of experiments obtained for the Corel-1k, Corel-10k and Caltech-256 datasets.

Wang et al. proposed a two-stage CBIR algorithm using sparse representation and feature fusion in which the global and local features are combined to retrieve the images [65]. The authors use the Generalized Search Tree resources for a first retrieval of images with similar scene information by measuring the Canberra distance. Afterwards, sparse coding and feature clustering are used to obtain the sparse representation of the local features extracted from the rough results of the retrieval. The similarity of the sparse feature vectors is measured through the Euclidean distance, obtaining the recovery results.

Regarding the Industrial Property context, one of the first research approaches found in the literature is to consider trademark images as a whole. The first example of application of this approach was the trademark system developed by Kato in 1992 [5]. In this system, the images were normalized to a grid of 8x8 pixels, and the Galois Field (GF) vectors for each image were calculated from the frequency of the distribution of the black and contour pixels by each cell of the grid. Subsequently, the comparison between images was made using the GF vectors. In the following years, several variants of this system were proposed, but with little significance in the success rate.

Another frequent approach in the literature consists of processing images as a discrete set of components and comparing them individually. From the comparison between the components, then, according to well-defined criteria, the global similarity between images is constructed. The first example of application of this methodology is the STAR system [34]. This system is based on the concept that the perception of similarity between commercial images is defined by the similarity of form, structure and semantics and that human intervention is indispensable to achieve acceptable results. The first processing stage involves manual indexing through which images are segmented according to the meaning of the components. In the second phase, manual indexing is complemented by the automatic indexing of the components, which contemplates the shape characteristics, through techniques such as Fourier descriptors or invariant moments; the structure, by identifying the presence of regular patterns of shapes and semantics, by identifying the presence of particular types of objects. The combination of the two indexing phases results in a very comprehensive characterization of the images, which then allows a high precision similarity assessment. However, this approach does not dispense with human intervention, and therefore it is associated with the same limitations as conventional approaches, namely the high delay and the propensity for errors.

Later, variants of this type of approach were presented in order to minimize or exclude human intervention in the process. An example of these is the ARTISAN system developed in 1998 [66], which differs from the STAR system in that it does not require human intervention in the process of indexing images. This was achieved by creating rules based on the principles of Gestalt Theory (or Theory of Form, a doctrine that argues that in order to understand the parts, it is necessary, first, to understand the whole [67]) to group the individual components of images in families of perceptual meaning. This system allows comparisons between images at three levels: global image, component families and individual components. Later versions of this system [68] incorporated multi-resolution analyses that allow extracting low-level characteristics from larger regions and that include a larger domain of shapes and structures. Other versions [69] focused on improving the application of Gestalt rules through the identification and grouping of all line segments with similar perceptual meaning.

Another technique based on the multiple possible interpretations of an image was developed in 2002 [70]. This system classifies the regions as solid or composed of lines, extracting from them the border contour and the skeleton of the drawing, respectively. After this processing of the images, the search is made by comparing the extracted line segments and the segments associated with the database images.

Jabeen et al. proposed a novel CBIR technique based on the visual words fusion of sped-up robust features (SURF) and fast retina keypoint (FREAK) feature descriptors [71]. The proposed system aims to adopt the strong points of the two aforementioned descriptors, combining the features of both systems to provide a good resistance to noise, detection errors and geometric and photometric deformations. The performance of CBIR is also improved due to the size of the dictionary in the case of visual words being twice as large when compared to features fusion, standalone SURF and standalone FREAK techniques.

Boia et al. [72] proposed a logo recognition system invariant to scaling, colors and illuminations coming from source lights of diverse intensities. The approach was based on the bag-of-words structure and scale invariant feature transform (SIFT) features. The authors note an improved accuracy in performance achieved through the complete rank transform feature when tested using the FlickrLogos-32 database.

### 2.2. Deep Learning Approaches

Deep-learning-based descriptor or hash code generation is the current trend of content-based large-scale image retrieval, mainly due to its computational efficiency and retrieval quality [73].

Related to algorithms and deep learning techniques, it is important to mention the development of the neuronal network AURA (Advanced Uncertain Reasoning Architecture), which consists of a family of generic techniques designed to perform search and correspondence operations in large unstructured databases [74]. This system has the Correlation Matrix Memory component at its core, which gives the network associative capacity.

Among the first works presented between the years 2011 and 2015 are the ones proposed by Krizhevsky and Hinton that used a deep autoencoder to map the images to short binary codes for CBIR [75], Kang et al. that proposed a deep multi-view hashing to generate the code for CBIR from multiple views of data by modeling the layers with view-specific and shared hidden nodes [76], Wu et al. that considered the multiple pretrained stacked denoising autoencoders over low features of the images [77] and Zhang et al., that developed a deep regularized similarity comparison hashing by training a deep Convolutional Neural Network (CNN) model to simultaneously optimize the discriminative image features and hash functions [78].

Most of the methods developed in this period use the resources learned by autoencoders and convolutional neural networks, facing problems in terms of less discriminative capacity, since, in general, the models are trained for the classification problem and the loss of information due to the quantization of resources. Since 2016, with the development of different network architectures and the design of several objective functions which lead to the high inter class separation and high intra class condensation in feature space, there has been a huge growth in image retrieval based on deep learning.

Varga et al. proposed an end-to-end supervised learning framework that learns probability-based semantic-level similarity and feature-level similarity simultaneously. Based on the results achieved using public datasets, the authors state that the developed hashing scheme is able to reduce the computational cost of retrieval significantly at the state-of-the-art efficiency level [79].

Bao et al. [80] experimentally investigated the appropriate architecture and settings of R-CNN for logo detection, using the FlickrLogos-32 database. The method compares several popular frameworks of R-CNN and considers the characteristics of logo objects in images.

Alzu’bi et al. proposed a CBIR system based on the use of a bilinear CNN [81]. In the proposed method, a CNN is used for the unsupervised extraction of features from the image without depending on the bounding boxes or annotation or any class label. The reduction of computational cost and memory usage is achieved by using the pooling scheme during the extraction process, and the extracted features are characterized by their reduced dimensions. The proposed approach was evaluated against large-scale image retrieval and showed good retrieval performance.

To improve the retrieval performance in terms of computational cost and memory usage, Tzelepi et al. [82] proposed a method where the feature representation is obtained through a CNN using maximum pooling after the convolutional layers rather than using the fully connected layers in order to overcome the discardless of the spatial information. With this approach, the authors reduce the dimension of the feature descriptor while retaining the spatial information, which results in high retrieval efficiency while requiring minimal memory storage and processing time. Despite a high accuracy, the method proposed by the authors does not employ an indexing technique, which requires more retrieval time.

The authors Singh et al. proposed a similar deep learning model applied to the CBIR on facial image data [83], where the activation of the convolution layer is used for feature representation including max-pooling as a feature reduction technique. The model uses partial feature mapping as image descriptor to incorporate the property of repeated information contained in facial images.

In order to reduce retrieval time, Sezavar et al. [84] proposed a CBIR algorithm that combines a CCN used to extract high-level features and as a classifier to specify the class of the query image and a sparse representation used to reduce the computational cost. Due to the use of a sparse representation, the method proposed by the authors has a lower precision than the one proposed by Tzelepi. However, the image retrieval is faster.

Zhang et al. proposed a CBIR with a convolutional Siamese neural network (CSNN) designed to to distinguish lung cancer from nodular/mass atypical tuberculosis in CT images [85]. In an initial step, the lesion patches are cropped out to compose lung cancer and nodular/mass atypical tuberculosis datasets, and the pairs of two arbitrary patches form a patch–pair dataset, which is used to train a CSNN. The distance between the query image and a fixed and predefined number of patches in both datasets is calculated using the trained CSNN. The patches closest to the query are used to give the final prediction by majority voting.

Recently, Monowar et al. proposed a deep convolutional neural network-based (DCNN) self-supervised image retrieval system [86]. The system is trained on pairwise constraints working in self-supervision, being able to be trained on a partially labeled dataset.

A comprehensive review submitted by Afshan Latif aims to reduce the semantic gap between image feature representation and human visual understanding, outlining recent developments in the area of CBIR and image representation, such as prominent image retrieval and representation models from low-level feature extractors to deep-learning approaches [87]. The review concludes that image feature representation is performed by the use of low-level visual features, such as color, texture, spatial layout and shape, making it impossible to be represented by using a single feature representation. A proposed solution revolves around the fusion of said low-level features, as they represent the image in the form of patches and as such, the performance is increased. The authors conclude that developing a large-scale image dataset for supervised training of a deep network is difficult and time-consuming and highlight the importance of performance evaluating deep networks.

Another more recent review aims its focus to the developments in the area of CBIR between 2009 to 2019, covering mainly the technological developments from the viewpoint of image representation and database search, summarizing the practical applications of CBIR in the fields of fashion image retrieval, remote sensing image retrieval and trademark image retrieval [32]. The authors also discuss future research directions of CBIR with the challenge of big data and the utilization of deep learning techniques.

Given the importance of preserving Industrial Property, in recent years some CBIR systems based on deep learning approaches specific to trademak images have been proposed.

Pinjarkar et al. [14] proposed trademark image retrieval through deep convolutional neural networks integrated with a relevant feedback mechanism, where the dataset features are optimized through particle swarm optimization, reducing the search space. At the preprocessing stage, the optimized features are clustered through the self-organizing map. In this proposal, the CNN model is trained on feature representations of relevant and irrelevant images using the feedback information from the user, bringing the marked relevant images closer to the query.

Trappey and Trappey [6] presented a methodology developed for trademark logo similarity measurement based on content-based image retrieval. The authors proposed a a transfer learning methodology that uses embedded learning with triplet loss to fine-tune a pre-trained convolutional neural network model. The results presented suggest a high accuracy, with a Recall@10 of the test set reaching 95%.

Cao et al. [88] proposed an unsupervised trademark retrieval method based on attention mechanism, adopting the instance discrimination framework and introducing a lightweight attention mechanism to allocate a more reasonable learning weight to key features. The authors state that the proposed method can obtain good feature representation of trademarks and improve the performance of trademark retrieval.

## 3. Methods

Due to the current nonexistence of a Content Based Image Retrieval System capable of overcoming the difficulties and requirements previously stated, in this paper, we propose a system built from the ground-up, integrating some popular machine learning concepts while also benefiting from proprietary advancements designed and implemented to improve results regarding trademark images.

### 3.1. System Description

The proposed method consists of a multi-phase implementation of different deep learning and image processing techniques which compose an image retrieval system based on deep learning principles. Depending on which of three possible inputs are submitted into the system, its functionality can vary slightly. Regardless, standard procedure is as described:

1. The submitted data data normalized to ensure proper reading and extraction in the following steps. In this scenario, normalizing an image means ensuring it conforms to a set of predefined rules. Images are resized to 224 resolution, and must contain a bit depth of 8 per color channel in the RGB format (24-bit depth total). Finally, images are converted to the jpeg format for faster processing and easier background recognition.
2. Upon preprocessing, a series of metadata information is extracted from the image. This information is stored and will improve filtering capabilities in a future process by allowing us to filter image data by additional parameters, including Nice classes, textual components and country of registration.
3. At this point, our image processing block starts extracting relevant features and components from images. There are two main algorithms at work here. First, we use a modified version of the VGG16 Classification Network [89], designed to extract a numeric representation of image features (feature vector). Secondly, we developed a hybridization algorithm centered around using image segmentation techniques, such as K-Means and Watershed, to extract individual significant visual components from images. This algorithm is fully documented in another paper we have published [90]. The combination of data regarding a specific image is then saved to a database as that specific image’s signature.
4. Upon applying user-defined filters, the algorithm computes a comparison pool comprised of all images that will be compared to the submitted data. This comparison process is achieved through the application of a mathematical formula specially develop to factor in neural network features and data regarding image components, such as its regions of interest. The result of this formula is guaranteed to be a number between 0 and 1, reflective of the similarity level found between two images, with 1 representing total visual equivalence (maximum similarity) between two images and 0 representing absolute visual discordance (maximum dissimilarity).
5. Results obtained in the previous step are further filtered according to post-search filters, usually reserved to setting a minimum threshold for two images to be regarded as visually similar and adequate for result presenting. This process results in much easier readability and faster result evaluating from the user. All presented results are also reversely ordered and organized into a performant python dataframe for easier indexing.

**Figure 1:**
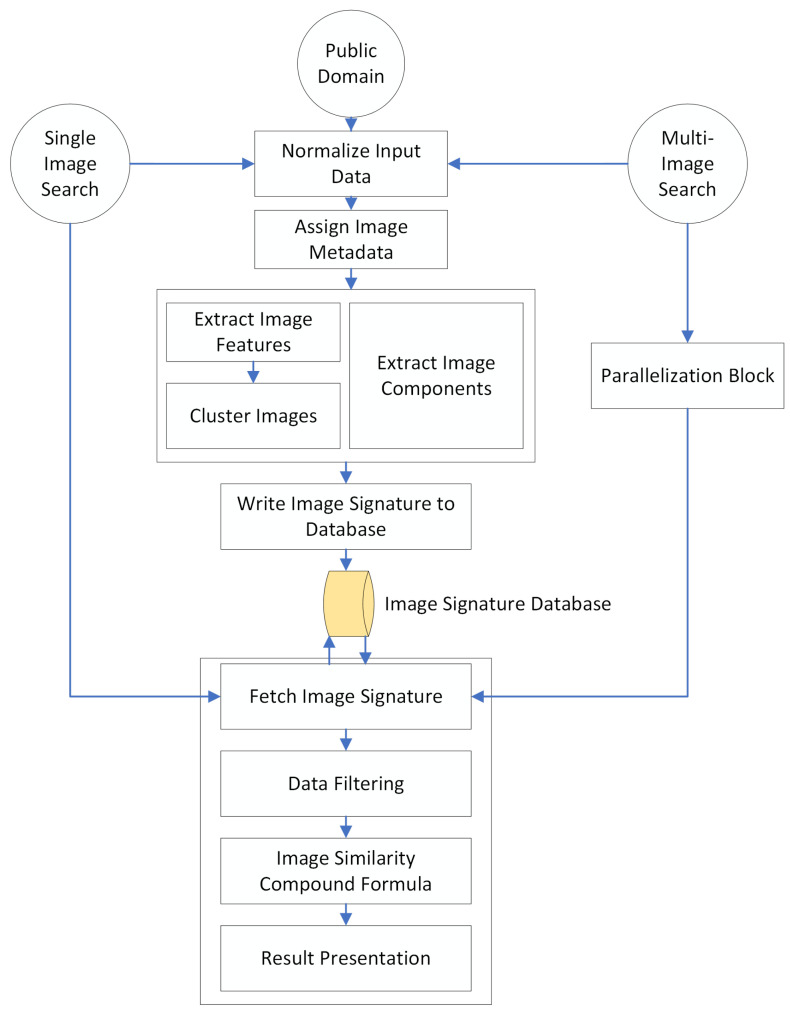
The Darwin graphical search engine workflow diagram.

Overall, the proposed method serves as a capable linear solution for all steps included in a typical CIBR system, namely image processing, image comparing and image retrieval. As a means to provide a better visualization of the system architecture, we present the proposed approach diagram in Figure 1. We also wish to provide insight to the building blocks that compose the described system. As such, the following subsections document some of the key features of our method as a means to contribute to the current state of the art in imaging systems.

### 3.2. Data Preprocessing

**Figure 2:**
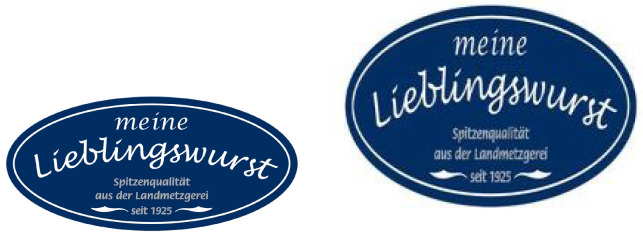
The process of resizing an image may affect its contents if original resolutions are rectangular.

Convolutional Neural Networks expect normalized input in order to correctly process an image. Due to these constraints, but to also aid in keeping information as constant as possible, we implement data pre-processing techniques in the earlier steps of our method. The network responsible for extracting relevant features from images expects input to be shaped as a **224×224×3** vector. As such, the first preprocessing step ensures this is the image shape that is used in the remaining steps of the algorithm. Additionally, it is important that images are coded with a bit depth of 24, as we wish to have 8-bit representations for the red, green and blue channels, working with the traditional $2^{24}$ color values used in digital RGB implementations. The downside of changing image resolution is that keeping aspect ratios consistent is not possible. As such, as illustrated in Figure 2, images that undergo heavier transformations, such as those with a rectangular resolution, may lose some semantic consistency in the process.

The original image on the left has a resolution of 1772×1181. When resized to 200×200, the long oval shape becomes much rounder and this, obviously, does not resemble the original shape at a 1:1 ratio. When performing transformative operations in such a large dataset, we believe that results are not negatively affected, and as such this does not constitute a problem in our system. Regardless, images are now adequately sized and ready to be processed by the image processing block rounded in green in the original diagram.

### 3.3. Parallel Computing

**Figure 3:**
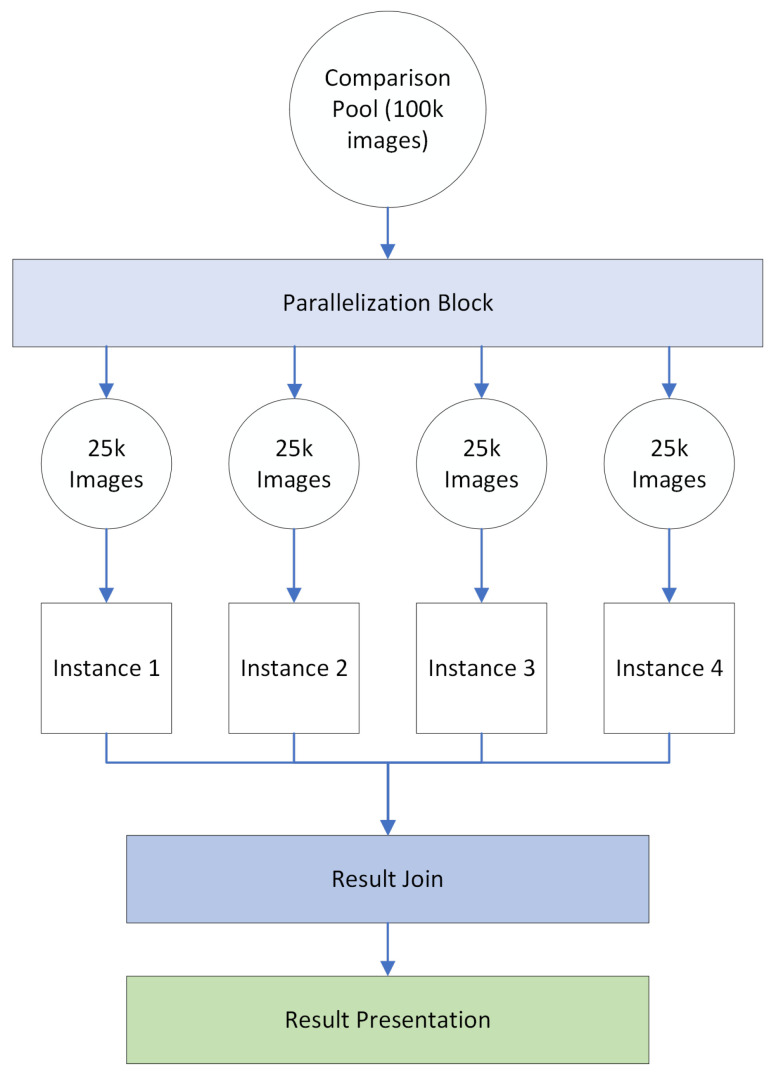
Multi-instance parallelization.

Allowing the user to perform multiple image searches at once comes with a significant performance disadvantage due to the high complexity of image signatures. As a means to mitigate this, we have developed a parallel processing block responsible for dealing with multi-image search scenarios. This parallelization is performed in one of two ways depending on the machine the system is deployed in. The first parallelization option is to simply compute the entire image comparison pool and then assign a portion of that pool to each system instance. For instance, if we want to split a 100,000 image comparison process, we can create four partitions of 25,000 images each and perform 4, individually instanced comparisons. In this case, we will have to join all the results and filter through them to present the final results to the user. A visual representation of this method and example can be seen in Figure 3. In this schema, the parallelization block is responsible by creating comparison pool subsets and running one parallel instance of the system for each subset generated, while the result join block is responsible by converting all four instance’s outputs into a singular view.

**Figure 4:**
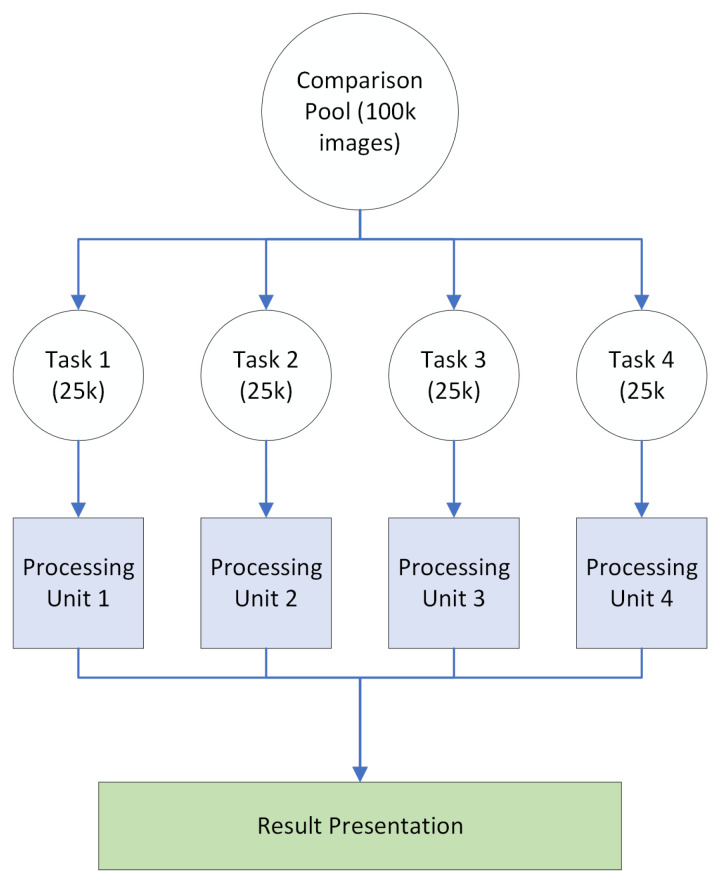
Multi-threaded parallelization.

Otherwise, parallelization can be performed on a single algorithm instance by assigning comparison blocks to multiple processors communicating via shared memory. Generally, this is the preferred method of parallelization as memory is more efficiently used, and system overloading is less prone to occur. This method, however, requires a multi-threaded machine for task assignment. A representation of this method can be seen in Figure 4. Note that because we are processing all data in a single instance of the system across shared memory, there is no need to compile results from the different processing units as each one has access to the other units’ results at all times.

### 3.4. Image Signatures

**Figure 5:**
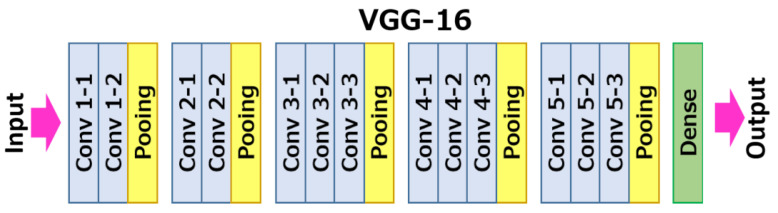
Headerless VGG-16 architecture.

**Figure 6:**
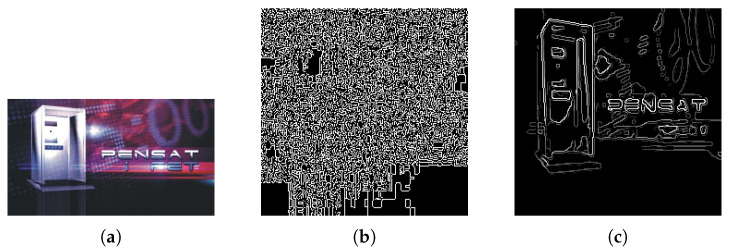
Differences between using default canny and simplified canny on a visually complex brand image: (**a**) original brand image; (**b**) default canny edge tracing; (**c**) K-Means clustering (K=4) + canny edge tracing.

We use the term image signature when referring to the set of data that describes a certain image’s composition and semantics as accurately as possible. The goal of an image’s signature is to provide us with a near exact representation of a certain image but with abstraction levels that allow us to compare it with other signatures as a means to achieve a fully capable image comparison process. In our system, an image’s signature is given by the following components:

- A 50-position feature vector extracted with a VGG16 transfer-learning convolutional neural network to which we removed the top 2 layers of the classification header, resulting in the architecture seen in Figure 5. To achieve the results we present onwards, there was no additional fine-tuning of the network as we do not perform any sort of classification on the images we process, but rather fully utilize the feature extraction capabilities of these deep neural networks. These features are originally represented by a 4096 position vector, later reduced to a more manageable and performant 50 positions through the application of the principal component analysis (PCA) dimensionality reduction algorithm.
- A 50-position edge feature vector achieved in a similar manner through the application of the network on a simplified canny edge version of the original image. Our canny algorithm contains an additional step responsible for applying four levels of K-Means clustering with the goal of consolidating similarly colored areas into more easily distinguishable shapes and blobs, resulting in very high quality edge representations in both clean and extremely noisy images. Figure 6 shows results when processing a visually dense and noisy image with a default canny implementation versus our canny implementation.
- The image’s K-Means assigned cluster computed with a centroid assignment function (K-Nearest Neighbors) immediately after feature extraction. A cluster label is used to quickly identify a set of images in the entire database that, by default, already contain a relatively similar feature vector. This is especially useful for performance reasons, as the overall length of comparison becomes far smaller.
- The image’s objects extracted with a hybrid K-Means and Watershed algorithm designed to automatically record object location, size and individual feature description [90]. This last component is specifically designed to improve algorithm robustness in cases where the overall image disposition may not be similar, but there may be an individual component on the image which automatically flags it as plagiarism. This attention to the image’s objects is specially useful in trademark image comparison due to enterprise’s tendency to resort to simpler graphical brand images rather than natural imagery.

### 3.5. Search Parameterization

The proposed system offers the ability to extensively filter search parameters, allowing the user to fine-tune the depth of search to perform. In our experiments, we found that the level of parameterization directly affects the time it takes to perform searches as the comparison pool size varies, with larger searches usually requiring more time to produce results. Filters belong to one of two categories. Pre-search filters affect the image collection which will be used to compare the input image to and are as follows:

- The assigned image cluster is the main pre-filter and search argument, directly reducing the image comparison pool. To avoid performing comparisons on the entire dataset, the system defaults to comparing the input image with the remainder of the images belonging to the same cluster.
- The 34 goods and 11 services class system known as Nice Classes can also be used to further reduce the comparison pool of the search algorithm. For instance, a user may only want to search for visually similar logos referring to companies registered in the same Nice classes.
- Similarly, the remaining image metadata, such as textual components, country of registration and Locarno classification can be used to specify searches regarding more or less information.

There is also a single Post-search filter which is applied after image similarities are computed. This filter acts as a threshold value for the minimum amount of visual correspondence needed for two images to be regarded as visually conflicting. By default, a minimum similarity value of 0.93 (or 93% correspondence) is required for a match to be considered. By either strengthening or weakening the filter complexity, there are benefits and downsides to the achieved results. We found that, intuitively, filter options which incur in a larger comparison pool provides more extensive search at the cost of processing time while narrowing down the subset of images to compare results in much faster, albeit more limited, comparisons.

### 3.6. Similarity Compound Formula

Because the goal of this system is to achieve image retrieval based on image similarity, we have developed a mathematical formula which always outputs a value between 0 and 1, with the former being two visually opposite images, while the latter represents two identical images. The proposed formula accounts for all components of an image signature and is calculated in a series of steps to simplify the operations. First, images are compared with regard to the objects they contain, and the formula used to compute object based similarity is as follows:

(1) $$S_{t}=\frac{\sum_{i=1}^{k}S_{i}}{K}-{\left(1-q\right)}^{2}\times w_{1}-{\left(1-j\right)}^{2}\times w_{2}$$

where *K* is the number of compared objects, *q* is the image space not taken up by objects (white space area), *j* is given by the Jaccard Coefficient between the number of objects in each image, and the $w_{i}$ values are simply weights for the two previous dissimilarity values. Finally, $S_{i}$ is the total similarity between any two images’ objects, given by:

(2) $$S_{i}=\frac{s\times w_{1}+r\times w_{2}+d\times w_{3}}{w_{1}+w_{2}+w_{3}}$$

where *s* is the individual similarity between two objects based on their feature vectors, *r* is the object proportion ratio between the larger object and the smaller object ($\frac{Area_{smaller}}{Area_{bigger}}$), *d* is the Manhattan distance between two object centers, and the $w_{i}$ values are independent (unrelated to Equation (1)) weights for each of the previous components, totalling 1. As previously mentioned, the end result of this formula is a single number, between 0 and 1 characterizing two image’s similarity level according to their objects. The output of this function is finally used in a third, final formula responsible for computing the final similarity score between two images.

(3) $$T_{ij}=\frac{O_{ij}\times w_{1}+F_{ij}\times w_{2}+E_{ij}\times w_{3}}{w_{1}+w_{2}+w_{3}}$$

where $O_{ij}$ refers to the image object similarity (Equation (1)) value, $F_{ij}$ is the Euclidean distance between image feature vectors, and $E_{ij}$ is the Euclidean distance between image edge features. These values are weighed so it is possible to assign a larger percentage to any component. Choosing the ideal weights for each component is a balancing act at its core, and thus it can be optimized by the usage of learning mechanisms. We discuss the usage of automatic weight optimization algorithms in the ’Future work’ section of the conclusion chapter as we intend to develop and write upon this concept.

## 4. Results

### 4.1. Dataset

The proposed method is meant to be used with individual user-submitted images for extraction and comparing. However, a large collection of public trademark images are collected daily, and this accounts for the comparison basis of our system (public domain input). At the date of writing this paper, this dataset is comprised of approximately 3,170,000 images belonging to 10 possible country jurisdictions with a wide spectrum of characteristics. Table 1 describes this dataset’s contents and features. In it, we have individually counted the number of images belonging to each of the processed jurisdictions and inspected the available image formats, as well as the minimum and maximum image resolutions encountered.

**Figure 7:**
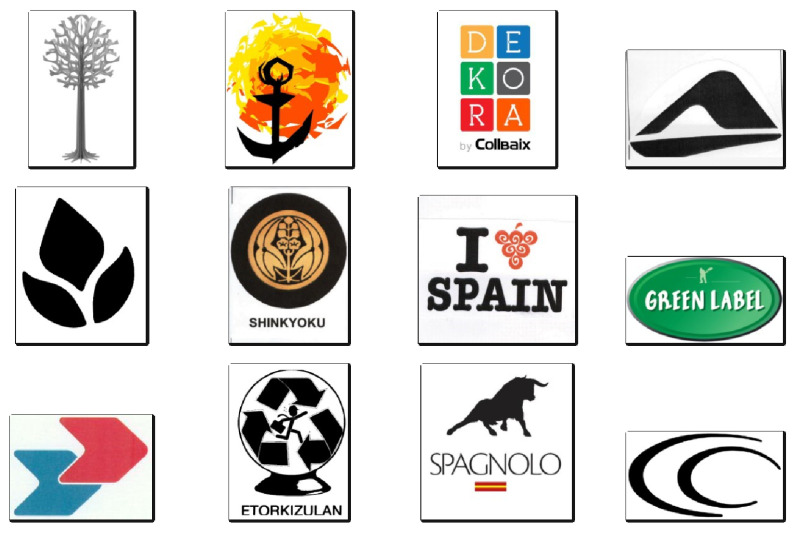
Trademark dataset 12-image sample.

A random selection of 12 images from varied jurisdictions is presented in Figure 7 to further characterize dataset contents. The border surrounding each trademark figure was added manually to visually aid in identifying image borders and demonstrate inconsistencies in image resolution and shapes.

### 4.2. Experimental Phase

**Figure 8:**
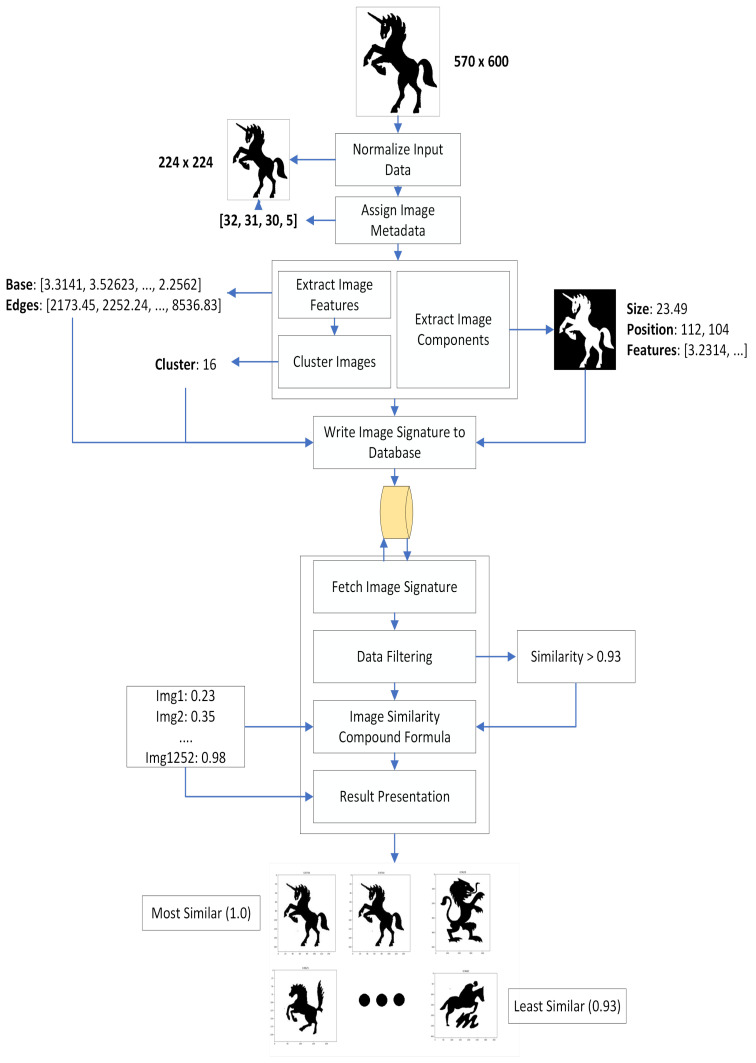
Image signature building and comparison process diagram.

As a means to present results demonstrative of the system capabilities, we planned an experimental phase which consisted of running a series of individual images through the algorithm and documenting achieved results throughout execution blocks. In Figure 8, we present a step-by-step demonstration of how a single image is processed and compared with a large dataset.

In the mentioned graphic, all documented steps are labeled with the data that is extracted or used in that specific step. The system begins by making sure the image fits perfectly within the input layer of the convolutional neural network, using predetermined metadata to make initial assumptions about the image contents, such as its likelihood of containing textual features rather than stylized visuals, which helps determine what the best comparison formula weights are. Next, all values pertaining to the image signature are extracted from the image and written to a database. This process can be either temporary, if we are dealing with a simple search and do not want data to be saved after presenting results, or permanent, if we wish to add the new image to the working dataset, making it available for usage in future searches. After an image signature is saved to the database, the system performs a series of matrix computing operations as a means to efficiently compute similarity values for a large amount of data.

### 4.3. Test Scenarios

**Figure 9:**
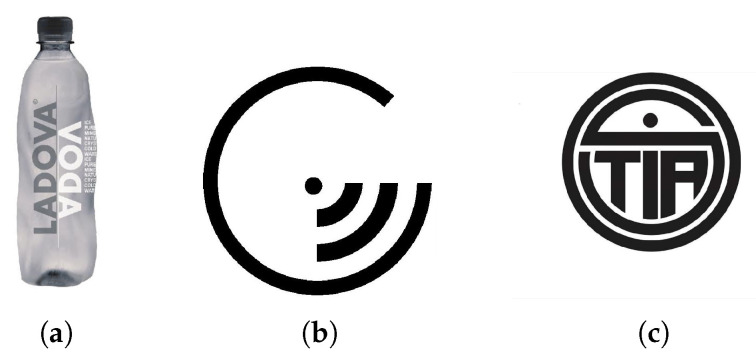
Selected test images used for algorithm retrieval efficiency evaluation: (**a**) input A; (**b**) input B; (**c**) input C.

**Figure 10:**
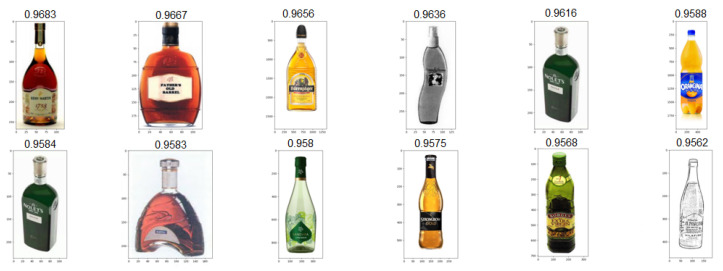
Top-12 result set for input A.

**Figure 11:**
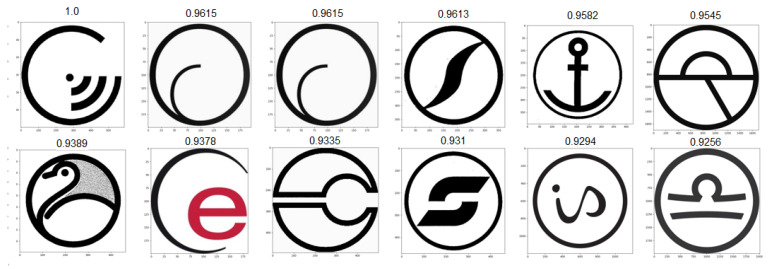
Top-12 result set for input B.

**Figure 12:**
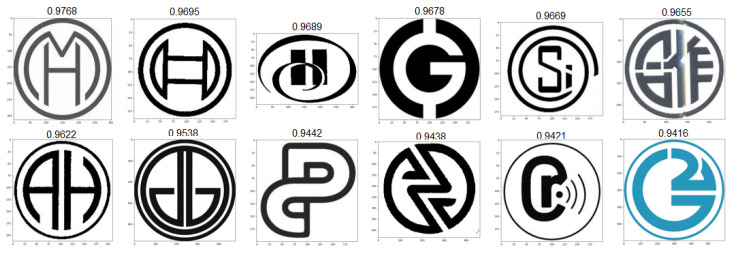
Top-12 result set for input C.

As a means to test our algorithm’s stability and result quality across a large variety of trademark images, we ran a series of 258 individual tests and evaluated results during all phases of the algorithm, from the initial edge conversion quality, to the quality of the final results. We strove for a good balance of variety, complexity and realistic imagery to account for the maximum possible scenarios. Below, we selected three result sets generated from images of varying complexity and style, presented in Figure 9. For computing all the similarity values present below, a standard weight vector of [0.35,0.40,0.25] was used for the feature, edge and object components, respectively. Adjusting component weights according to expected image contents (for example, adjusting one specific weight vector for each image cluster), is likely an optimal way of ensuring the best results across all image types. For the sake of analysis, we kept these values consistent. Our first input, A, represents a case where brand imaging resorts to using a real product picture, in this case a bottle. Despite it not being the intended input for the proposed system, results showcase good adaptability to real images, with all 12 resulting images in Figure 10 also resembling bottles of various shapes and sizes. This specific result set is especially useful to showcase the level of abstraction we can achieve by processing images with convolutional neural networks, as they work exceptionally well at extracting relevant features from known day-to-day objects. Our next proposed input, B, is intended to demonstrate the edge processing component of our system. In recent years, brands have been using more simplistic approaches to their trademark images, often resulting in minimal sets of lines and shapes, which can all be easily converted into edge representations for excellent readability for our network. The resulting set presented in Figure 11 is totally composed by similarly black and white round logos with some sort of minimal design embedded in the circular shape. Upon closer inspection, we observed that in this case the edge-based similarity value was responsible for the selection of over 73% of the result images, meaning the remaining two components (features and objects) would not have been enough to correctly retrieve similar images in scenarios such as this one. Additionally, it is relevant to mention that the second and third results are apparently identical. This is not a system fault and is instead caused by that same image being registered in two completely different country jurisdictions, resulting in a duplication of the image in the dataset. Finally, input C is a test intended to directly challenge the system to process an image that appears to be relatively simple but actually contains intricate designs encased in a round shape, resembling a more complicated version of input B. The resulting image set shown in Figure 12 is more diverse than the previous ones, likely due to algorithm limitations or simply a lack of more similar images in our dataset. Regardless, most result images correctly display round logos with some sort of heavily stylized typography component in the middle. The main particularity of this last set is that all three similarity components have heavily impacted the formula results, meaning there was not a single component responsible for the vast majority of all result selections. In the following results, we have manually enhanced the similarity value text presented above each result, allowing for better readability.

With the demonstration and discussion of these three search cases, we figure it is also important to mention the elapsed time for each processing phase, as one of the most important requirements for a good image retrieval system is the time it takes to retrieve results. As such, Table 2 showcases the individual processing times for different processing phases in the presented result sets and the average processing times for the remainder of the quality control tests. We have also included a column which represents the total comparison pool for each test, keeping in mind all tests were performed on default settings, meaning we have only searched in the image cluster our input image belongs in.

### 4.4. System Performance Evaluation

Performance metrics are widely used in information retrieval systems to evaluate the quality of the data retrieved and are usually measured in relation to a ground-truth value, such as predefined labels of a certain dataset. In cases such as this one, when working with completely unlabelled data and having performed zero training steps on the utilized network, evaluating performance metrics can be challenging. Generally, the precision, recall, F-Score and mean average precision (mAP) measures are used when evaluating image retrieval systems. Precision, defined by Equation (4), is the percentage of correctly retrieved images, also known as true positives (TP), out of the total number of retrieved images, given by the sum of true positives and false positives (FP).

(4) $$P=\frac{TP}{TP+FP}$$

Recall, defined by Equation (5), is the percentage of correctly retrieved images TP out of the total number of relevant images present in the dataset, given by the sum of true positives (TP) and false negatives (FN).

(5) $$R=\frac{TP}{TP+FN}$$

F-score, which describes the accuracy of the image retrieval system, is the harmonic mean of precision and recall, defined as:

(6) $$F=\frac{2\times P\times R}{P+R}$$

Mean Average Precision (mAP) is the mean value of the Average Precision (AP) of all the queries, where the definition of *AP* for the *i*th query is formulated as follows:

(7) $$AP_{i}=\frac{1}{Q_{i}}\sum_{n=1}^{N}\frac{R_{i}^{n}}{n}t_{n}^{i}$$

where $Q_{i}$ is the total number of relevant images for the *i*th query, *N* is the total number of images of the search set, $R_{i}^{n}$ is the number of relevant retrieved images within the *n* top results, and $t_{n}^{i}$ is an indicator function, being equal to 1 if the *n*th retrieved image is relevant to the *i*th query, and 0 otherwise.

Table 3 presents the performance metric values obtained for inputs A, B and C.

As previously mentioned, our dataset is comprised of over 3,000,000 images and grows daily. As a means to work around the fact that all data are unlabelled and thus precise performance evaluation is prone to bias, the assessment of the relevance of the results returned by the proposed system was carried out by a group of unbiased users from different countries and professional backgrounds. Each of the users was tasked with manually looking through reduced sections of the dataset and tagging images that they consider relevant for either of the query images presented. This process took place over the course of three days and produced a relatively complete baseline for what results are expected by the system.

### 4.5. Comparison with State-of-the-Art CBIR Systems

Efficient system performance evaluation requires appropriate comparison with systems developed with a similar goal in mind. In Table 4, we compare the proposed system with other CBIR systems developed in the past few years. As a means to keep comparisons fair and relevant to the objective of each system, a short system description, as well as the utilized techniques and dataset dimensions are all included in the table.

When evaluating the meaning of the presented results, it is important to note that the complete lack of classes in our dataset is a differentiating factor of our system when compared to others, as every image is unlabelled, and thus there are no preset ground-truth values to accurately measure performance against. Additionally, dataset dimensions for other systems developed in this area contain only a fractional amount of the images in our dataset, making human-assigned ground-truth labels an extremely time-consuming process with no added benefits. To potentially affect achieved results in a positive way, our system will benefit greatly from continuous use and the implementation of feedback systems which will evaluate results and optimize system hyperparameters based on user interaction.

## 5. Conclusions and Future Work

In this paper, we intended to document the work developed in the sense of building a novel content-based image retrieval system based on deep learning techniques capable of overcoming the difficulties associated with having a highly diversified trademark image dataset and also expanding upon concepts and ideas proposed by other authors who have published in the related field, implementing multi-feature weighing, semantic evaluation of images and building an overall robust system capable of processing and comparing most image types. The proposed approach has linear complexity, and despite its inert complication due to the involvement of deep convolutional neural networks, it presents processing times that are compatible with real-time applications. The experiments performed aim to showcase several levels of image complexity and contents, and the corresponding results show adequate image retrieval capabilities, displaying a clear and undisputed similarity between input and output, both on a semantic basis and a graphical basis. Implemented adjustments in the proposed system allow for even greater result filtering, and we believe the ever-growing nature of the comparison dataset will only further improve result efficiency and accuracy. To improve user satisfaction and increase accuracy of the proposed system, we aim to include a relevance feedback stage, which requires user intervention to specify relevant and irrelevant retrieved images. The system updates or re-weights the representation and metrics, and then recalculates the revised results. This stage is a challenging task that needs more attention to achieve maximum user satisfaction with minimal iterations.

## Figures and Tables

**Table 1:** Dataset characterization table.

Jurisdiction	Image Count	Formats	Resolution
Europe	682,009	.JPG, .PNG	140 × 140 to 4128 × 2322
Portugal	238,893	.JPG	256 × 144 to 5000 × 5000
World	549,673	.JPG, .PNG	90 × 90 to 4200 × 4200
Angola	44,033	.JPG	128 × 128 to 1024 × 1024
Moçambique	200,990	.JPG	128 × 128 to 1024 × 1024
Cabo Verde	2713	.JPG	50 × 50 to 2000 × 2000
Brazil	434,456	.JPG, .PNG	64 × 64 to 4096 × 4096
Spain	989,791	.JPG, .PNG	128 × 128 to 5000 × 5000
São Tomé e Príncipe	5332	.JPG	128 × 128 to 1024 × 1024

**Table 2:** Average system execution time for the 258 image test scenario.

DarwinGSE Processing Times (in Seconds)					
**Test Sample**	**Signature Extraction**	**Database Querying**	**Pool Size**	**Comparison**	**Total**
Input A	3.34	0.14	64,530	0.84	4.32
Input B	2.88	0.19	92,358	1.02	4.09
Input C	3.17	0.12	54,362	0.63	3.92
Average	3.61	0.23	-	0.75	4.36

**Table 3:** Proposed System Performance.

Input	TP	FP	FN ${}^{\left(1\right)}$	Precision	Recall	F-Score	AP
A	459	23	12	95.2%	97.4%	96.1%	95.7%
B	71	27	8	72.4%	89.8%	80.2%	73.1%
C	132	9	7	93.6%	94.9%	94.2%	93.9%

^(1)^ Estimated values acquired through a series of human tests.

**Table 4:** Proposed System comparison with state-of-the-art deep learning CBIR systems.

System	Dataset	Size	Classes	Algorithm	mAP
Darwin (ours)	Global Brand Database	$3.1\times 10^{6}$	Unlabelled	Combined Multiple Features	93.7%
Tzelepi and Tefas [82]	Paris-6k [91]	6392	11	Model Retraining for Compact Descriptors	$83.47\%{\;}^{\left(1\right)}$
Monowar et al. [86]	CIFAR-10 [92]	$6\times 10^{4}$	10	Self-Supervising and Recurrent Networks with Spatial Pooling	83.5%
Alzu’bi et al. [81]	Oxford	5062	11	Bilinear Root Compact Pooling of Deep Convolutional Features	88.6%

^(1)^ Value achieved for the unsupervised model.

## Data Availability

The data presented in this study is available on request from the corresponding author. The data are not publicly available due to company privacy matters, however, all data contained in the dataset mentioned in the manuscript is publicly available.

## Abbreviations

The following abbreviations are used in this manuscript:

CBIR	Content Based Image Retrieval
CNN	Convolutional Neural Network
DCNN	Deep Convolution Neural Network
RGB	Red, Green, Blue
